# Myostatin silencing inhibits podocyte apoptosis in membranous nephropathy through Smad3/PKA/NOX4 signaling pathway

**DOI:** 10.1515/med-2022-0615

**Published:** 2023-03-23

**Authors:** Juan Wang, Bangjuan Shang, Li Tang, Min Tian, Junping Liu

**Affiliations:** Department of Nephrology, Xianyang Central Hospital, Xianyang, Shaanxi Province, 7120000, China; Department of Nephrology, Xianyang Central Hospital, No. 78 East Renmin Road, Weicheng District, Xianyang, Shaanxi Province, 7120000, China

**Keywords:** membranous nephropathy, myostatin, Smad3, protein kinase A/NADPH oxidase 4 signaling pathway

## Abstract

This article focuses on deciphering the effect of myostatin (MSTN) on podocyte apoptosis in membranous nephropathy (MN) and fathoming out its underlying mechanism. Rats received the intravenous injection of cationized-bovine serum albumin to induce MN *in vivo*, while angiotensin II (Ang II) was exposed to AB8/13 cells to induce MN model *in vitro*. The mRNA expression of MSTN was detected by qRT-PCR. The effects of MSTN silencing on MN model rats and cells were assessed by cell counting kit-8 assay, flow cytometry, hematoxylin and eosin staining, and TUNEL assay. The expressions of proteins related to apoptosis and Smad3/protein kinase A (PKA)/NADPH oxidase 4 (NOX4) signaling pathway were examined by western blot. As a result, MSTN was highly expressed in MN cell and rat models. Besides, knockdown of MSTN elevated the MN cell viability and dwindled apoptosis rate, as well as attenuated kidney injury in MN rats. Meanwhile, MSTN silencing lessened the expressions of phosphorylated (p)-Smad3 and Nox4, while boosting the p-PKA expression in MN rats and cells. Additionally, Smad3 overexpression reversed the above effects of MSTN silencing on Ang II-induced podocytes. In conclusion, MSTN knockdown restrains the podocyte apoptosis through regulating Smad3/PKA/NOX4 signaling pathway.

## Graphical abstract

**Figure:**
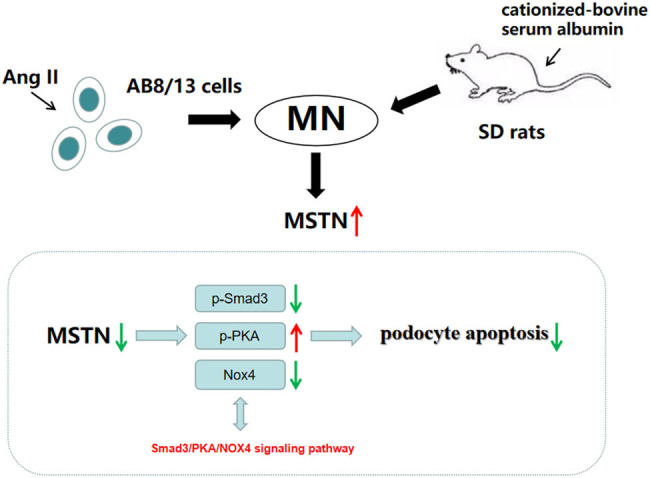

## 1 Introduction

Membranous nephropathy (MN) is one of the most common pathological types of adult nephrotic syndrome [1]. With diffuse basement membrane thickening, the immune complex primarily composed of immunoglobulin G and complement is deposited under the glomerular basement membrane (GBM) epithelial cells, which is the main pathological feature of MN [2]. However, the pathogenesis of MN is still vague. Most scholars believe that MN is the glomerular injury against podocyte membrane antigen mediated by autoantibodies, which could eventually elicit renal failure [3]. The duration of MN is lengthy, and renal function damage often occurs 5–10 years after the onset. As such, early diagnosis and treatment of MN play an important role in preventing or delaying its deterioration [3]. Renal biopsy has long deemed as the gold standard for the diagnosis of MN, but it is a traumatic operation, which may lead to serious bleeding complications. In addition to that, renal biopsy also has some contraindications in clinic, such as solitary kidney, psychosis, severe hypertension, etc. [4,5]. These limitations of renal biopsy restrain its wide application in MN. In view of this, it is of great practical significance to find safe, rapid and effective non-invasive indicators for MN diagnosis and treatment.

According to the previous study [6], GSE73953 dataset containing microarray data from MN patients and healthy controls was analyzed to obtain different expressed genes, of which the myostatin (MSTN) gene with the highest expression aroused our great interest. MSTN, located at 2q32.2 in the human genome, is a growth- and differentiation-related factor [7]. The MSTN gene has three putative transcription initiation sites, and is transcribed as a 3.1-kb mRNA species that encodes a 375-aa precursor protein [8]. In addition, it is expressed uniquely in the human skeletal muscle as a 26 kDa mature glycoprotein and secreted into the plasma, which binds to the receptor with the systemic circulation, resulting in a series of biological effects [8,9]. For instance, mature MSTN dimer phosphorylates type I receptors (ALK4 and ALK5), can bind to type II receptors ActRIIB and ActRIIA, and then promote the phosphorylation of Smad2 and Smad3, while phosphorylated (p)-Smad2 and p-Smad3 can form complexes with Smad4 and transfer into the nucleus, thereby activating the transcription and expression of atrophy genes by interacting with DNA and other nuclear factors [10,11]. The current study shows that the inhibition of MSTN has a good therapeutic effect on chronic kidney disease and the expression of MSTN may be associated with renal function [12,13]. However, the detailed role of MSTN in MN requires further exploration.

It has been proved that MSTN is able to activate p-Smad3 [14], and high glucose (HG)-induced increase in p-Smad3 level as well as Smad3-mediated PKA/NOX4 signaling pathway are important causes of podocyte apoptosis [15]. Therefore, this study is committed to further probing into whether MSTN is responsible for advancing podocyte apoptosis in MN through the Smad3/PKA/NOX4 pathway, and investigating the underlying mechanism of MSTN in MN.

## 2 Materials and methods

### 2.1 Animals and ethics statement

A total of 40 Sprague-Dawley rats (8–10 weeks) were purchased from Jiangsu ALF Biotechnology (China) and housed in cages. The light duration followed a normal circadian rhythm, room temperature was maintained at 22 ± 2°C, and humidity was set at 45–50%, with food and water supplied *ad libitum*. All animal experiments were performed in Xianyang Central Hospital, on the premise of complying with the guidelines of the China Council on Animal Care and Use and acquiring the approval from the Committee of Experimental Animals of Xianyang Central Hospital (S202008019).

### 2.2 Bioinformatics assay

GSE73953 dataset was downloaded from the Gene Expression Omnibus database (https://www.ncbi.nlm.nih.gov/geo/), which included peripheral blood mononuclear cell samples of IgA nephropathy, MN, and healthy controls. The “limma” R package was used to analyze the samples of MN (*n* = 8) and healthy controls (*n* = 16) in the GSE73953 dataset, and then the volcano map and heat map were acquired. The gene functional enrichment and signaling pathway enrichment of upregulated genes were analyzed by Gene Ontology (GO, http://geneontology.org/) and Kyoto Encyclopedia of Genes and Genomes (KEGG, https://www.kegg.jp/).

### 2.3 Cell culture

Podocyte cell line AB8/13 (152135, Ximbio, UK) was cultured in RPMI 1640 medium (PM150110, Procell, China) supplemented with 10% fetal bovine serum (164210-500, Procell) and 1% penicillin–streptomycin solution (PB180120, Procell) at 37°C with 5% CO_2_.

### 2.4 Cell transfection

Smad3 overexpression plasmid was constructed using pcDNA3.1 (V79520, Invitrogen, USA). Then, Smad3 overexpression plasmid, empty vector (negative control, NC), small interfering RNA (siRNA) targeting MSTN (siMSTN, siG000002660A-1-5, Ribobio, China), and siRNA negative control (siNC, siN0000001-1-5, Ribobio) were transfected into AB8/13 cells by Lipo6000 transfection reagent (C0526, Beyotime, China) according to the supplied instruction. Later, the successful transfection was confirmed by quantitative real-time polymerase chain reaction (qRT-PCR) and western blot.

### 2.5 Establishment of MN model and grouping

An *in vivo* MN rat model was established by the induction of cationized-bovine serum albumin (c-BSA) with reference to the previous methods [16]. Briefly, c-BSA (BSA; 23210, Thermo Scientific, USA) was prepared according to Border’s method [17], and then each rat was intravenously injected with 50 mg/kg of c-BSA that had been dissolved in 0.5 mL 0.01 M phosphate-buffered saline (PBS; PB180327, Procell, China) through the tail vein every day for 14 days. Thereafter, c-BSA-induced MN model rats were injected with 30 pmol/g of short hairpin RNA targeting MSTN (sh-MSTN, GACTGTACATGCATTAAAATTTT) or shRNA negative control (sh-NC) lentivirus that were constructed by pGLVU6/GFP lentiviral interference vector (C06001, Genepharma, China) via tail vein [18]. All rats were divided into the four groups: control group (rats received equal volume of PBS), model group (c-BSA-induced MN model rats), model + sh-NC group (model rats were injected with sh-NC via tail vein), and model + sh-MSTN group (model rats were injected with sh-MSTN via tail vein), with ten rats in each group. Ten days after c-BSA induction, rats were sacrificed by cervical dislocation to obtain the kidney tissues.

An *in vitro* MN cell model was established by stimulating AB8/13 cells with 100 nmol/L of Angiotensin II (Ang II, 4474-91-3, MedChemExpress, China) for 24 h [19]. All cells were grouped into two parts. The groups in the first part were as follows: control group (normal cells), model group (Ang II-treated cells), model + siNC group (siNC-transfected cells were treated with Ang II), and model + siMSTN group (siMSTN-transfected cells were treated with Ang II). In the second part, there were four groups: model + siNC + NC, model + siMSTN + NC, model + siNC + Smad3, and model + siMSTN + Smad3 groups. In the model + siNC + NC and model + siMSTN + NC groups, Ang II was administrated on cells transfected with siNC/siMSTN and NC, while in the model + siNC + Smad3 and model + siMSTN + Smad3 groups, cells were transfected with siNC/siMSTN and Smad3 overexpression plasmid, and then exposed to Ang II.

### 2.6 Hematoxylin and eosin (H&E) staining

The partial kidney tissues of rats were fixed in 4% paraformaldehyde (AR1068, Bosterbio, USA) at room temperature overnight, then dehydrated and embedded in paraffin, and sectioned in serial cross-sections. Next, the 5 µm thick sections were successively stained with hematoxylin (H3136, Sigma-Aldrich, USA) and eosin (E4009, Sigma-Aldrich, USA). Ultimately, the photomicrograph of the stained sections was captured under a microscope (×200, ×600, Leica Microsystems, Germany).

### 2.7 Terminal deoxynucleotidyl transferase-mediated dUTP nick end labeling (TUNEL) assay

The apoptotic cells in rat kidney tissues were identified using TUNEL kit (40306ES20, Qcbio, China) according to the supplied protocol. In short, rat kidney tissue sections were immersed in 4% paraformaldehyde and PBS at room temperature for 15 min, respectively, and then permeabilized with proteinase K for 10 min. Thereafter, the tissue sections were incubated with TUNEL reaction buffer at 37°C for 2 h, and then counterstained with WT-1 staining to visualize podocytes. Eventually, the TUNEL-positive cells and WT-1-positive cells were visualized by Olympus CX43 microscope (Japan) at ×200 magnification. A total of 50 glomeruli per kidney were calculated.

### 2.8 QRT-RCR

Total RNA of cells and rat kidney tissues was extracted by Triquick Reagent (R1100, Solarbio, China), and reversely transcribed to cDNA using Universal RT-PCR Kit (RP1105, Solarbio, China) according to the manufacturer’s instructions. MSTN expression was determined by SYBR Green PCR Mastermix (SR1110, Solarbio, China) using an ABI7900-HT-Fast device (Applied Biosystems, USA) in according with the protocols of manufacturer, with glyceraldehyde-3-phosphate dehydrogenase (GAPDH) serving as the endogenous control. Subsequently, the data calculation was performed by the 2^−ΔΔCT^ method [20]. The sequences of the reverse (R) and forward (F) primers are listed from 5′ to 3′: MSTN, (F) GGCATGGTAATGATTGTTTCCGTG, (R) TTTACCTGTTTGTGCTGATTGCTGC; GAPDH (F) AAATGGTGAAGGTCGGTGTGAAC, (R) CAACAATCTCCACTTTGCCACTG.

### 2.9 Cell counting Kit-8 (CCK-8) assay

The 5 × 10^3^ cells per well were inoculated into 96-well plate. 24 and 48 h post-transfection, 20 μL CCK-8 reagent (E606335, Sangon, China) was added to each well and incubated the cells for an additional 4 h. The final absorbance was tested by a microplate reader (VL0000D2, ThermoFisher, USA) at a wavelength of 450 nm.

### 2.10 Flow cytometry

The cell apoptosis was tested by Annexin V-FITC/propidium iodide (PI) Apoptosis Detection Kit (A211-01, Vazyme, China). In brief, AB8/13 cells were plated in a six-well plate and cultured for 48 h. Afterward, the cells were washed with binding buffer and centrifuged at 1,300 rpm for 3 min. Thereafter, the cell pellet was resuspended in 200 μL of binding buffer. Finally, the cells were stained with 5 μL Annexin V-FITC and PI in the dark for 15 min and detected by a CytoFLEX flow cytometer (Beckman Coulter, USA).

### 2.11 Western blot

The total protein of cells and rat kidney tissues was extracted with RIPA Lysis Buffer (C500007, Sangon, China), and the protein concentration was determined using BCA assay kit (C503021, Sangon, China). An equivalent of 30 μg protein extract and 5 µL marker (PR1910, Solarbio, China) were resolved on the SDS-PAGE, followed by being transferred onto PVDF membrane (IPFL00010, Millipore, USA). Next, the protein blot was blocked with skim milk, and probed with primary antibodies, followed by further incubation with appropriate secondary antibody goat anti-rabbit IgG (1:2,000, ab7090; abcam, UK). Subsequently, immunoreactive bands were detected using an ECL kit (ab133409; abcam). GAPDH served as an internal control. The protein bands on X-ray films were quantified with a Tanon 5200 imaging system (Tanon, China). The primary antibodies from abcam and Cell Signaling Technology (CST) are listed as follows: Bax (1:2,000; Rabbit; ab182733, 21 kDa; abcam), Bcl-2 (1:500; Rabbit; ab194583, 26 kDa; abcam), Cleaved Caspase 3 (1:1,000; Rabbit; #9661, 17 kDa; CST), p-Smad3 (1:2,000; Rabbit; ab52903, 48 kDa; abcam), Smad3 (1:1,000; Rabbit; ab40854, 48 kDa; abcam), p-protein kinase A (p-PKA, 1:1,000; Rabbit; #5661, 42 kDa; CST), PKA (1:1,000; Rabbit; #4782, 42 kDa; CST), NADPH oxidase 4 (Nox4, 1:1,000; Rabbit; ab154244, 67 kDa; abcam), and GAPDH (1:10,000; Rabbit; ab181602, 36 kDa).

### 2.12 Statistical analysis

Statistical analysis was performed using GraphPad Prism 8.0. The measurement data were expressed as mean ± standard deviation. One-way analysis of variance was adopted for multiple group comparisons, and Bonferroni test was applied for further analyses. Differences with *p* < 0.05 were considered to be statistically significant.

## 3 Results

### 3.1 MSTN was an upregulated gene in MN and was related to apoptosis

**Figure 1:**
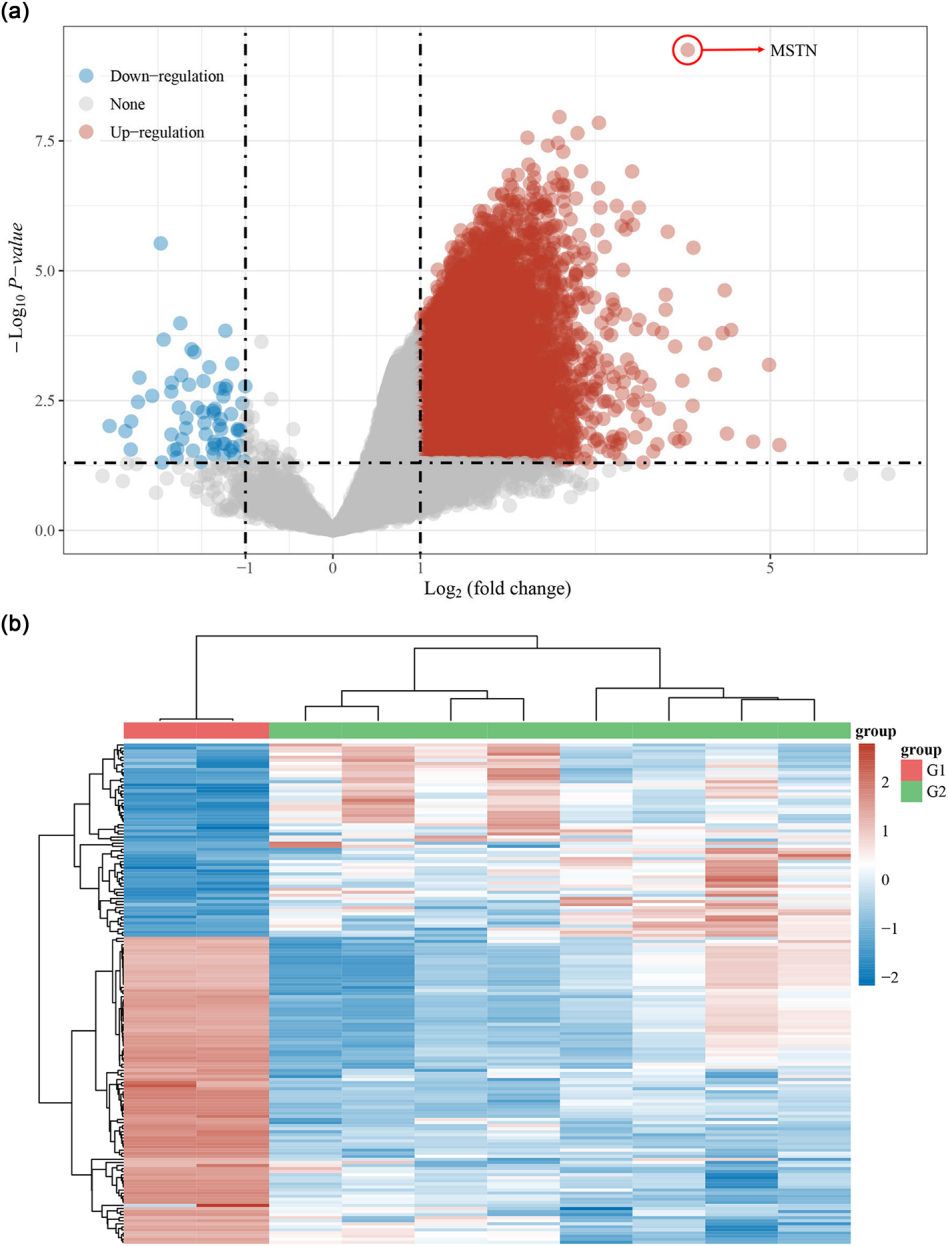
Differentially expressed genes in GSE73953 dataset. (a) Volcano map of differentially expressed genes in GSE73953 dataset. Fold change represents the degree of gene upregulation or downregulation in the dataset, while the dash-dotted lines are used to distinguish the genes (|logFC| > 1). *p* < 0.05. (b) Heat map of differentially expressed genes in GSE73953 dataset. G1 group represents the peripheral blood mononuclear cells from 16 healthy controls, while G2 group refers to peripheral blood mononuclear cells from eight membranous nephropathy patients. Each row denotes different samples in G1 and G2 groups, and each column represents differential genes in the G1 and G2 group.

**Figure 2:**
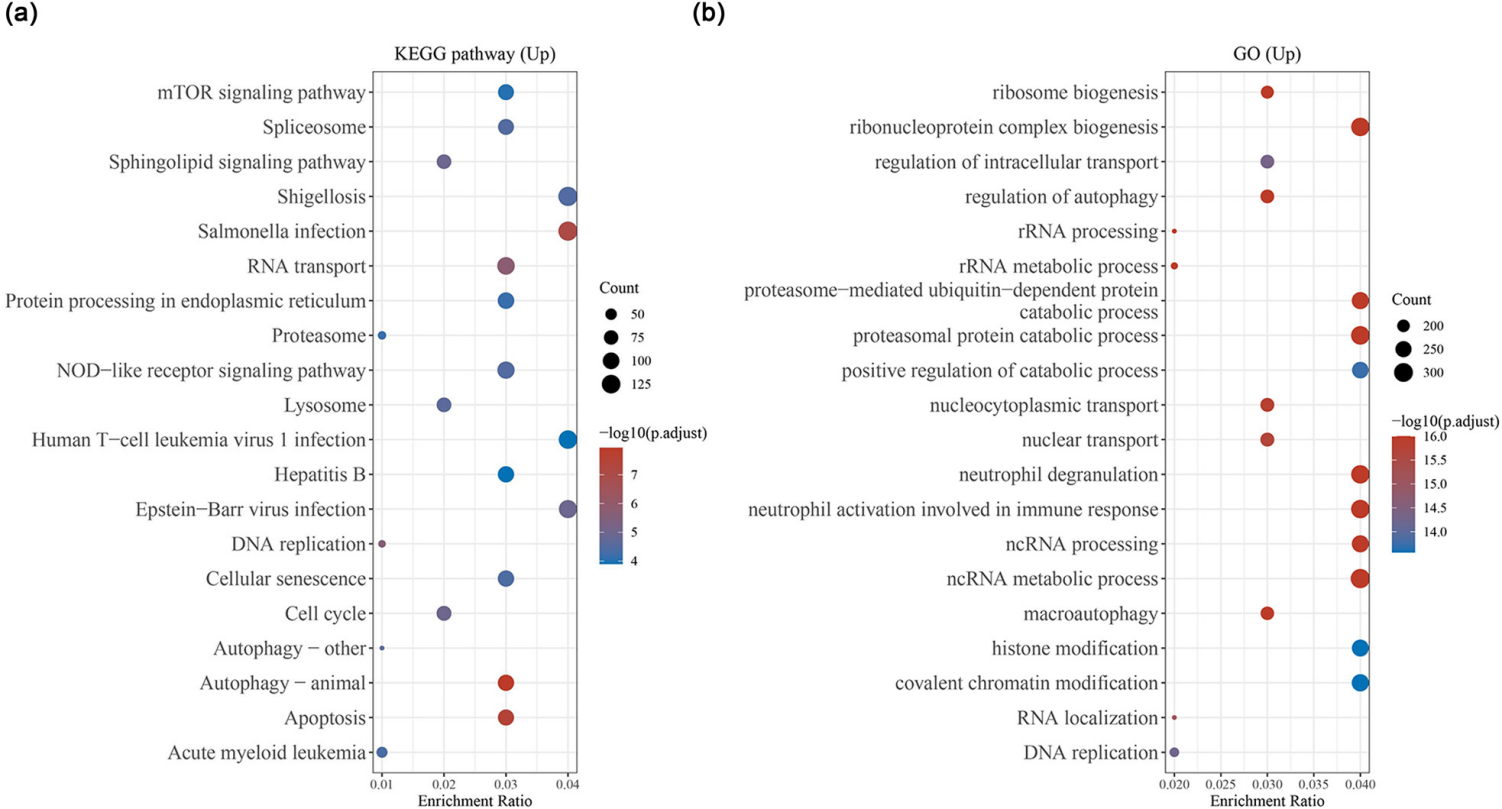
KEGG and GO analyses of upregulated genes in GSE73953 dataset. (a) KEGG analysis of signaling pathway enrichment in upregulated genes (https://www.kegg.jp/). (b) GO analysis of upregulated gene functional enrichment (http://geneontology.org/). Abbreviation: KEGG, Kyoto encyclopedia of genes and genomes; GO, gene ontology.

Through the analysis on GSE73953 dataset comprising of microarray data from MN patients and healthy controls, we discovered from the volcano map that a large number of aberrantly expressed genes met the screening conditions (|logFC| > 1, *p* < 0.05, Figure 1a). In addition, the heat map (Figure 1b) displayed the differential genes in peripheral blood mononuclear cells from healthy controls (G1 group) or MN patients (G2 group). Combined the results of these two figures, it could be observed that MSTN presented the most obvious upregulation (Figure 1). Meanwhile, the upregulated genes in GSE73953 were analyzed by KEGG and GO. It turned out that upregulated genes were closely related to the biological processes including autophagy (animal) and apoptosis (Figure 2a). In addition, the gene enrichment ratio was remarkably increased during the ribonucleoprotein complex biogenesis, neutrophil degranulation, ncRNA metabolic process, and other processes (Figure 2b). Based on the above results, it could be concluded that MSTN with upregulated expression in MN may be related to autophagy, apoptosis, and other biological changes. Accordingly, we hereby focused on its effect on apoptosis.

### 3.2 Silenced MSTN alleviated Ang II-induced AB8/13 cell injury via regulating Smad3/PKA/Nox4 signaling pathway

**Figure 3:**
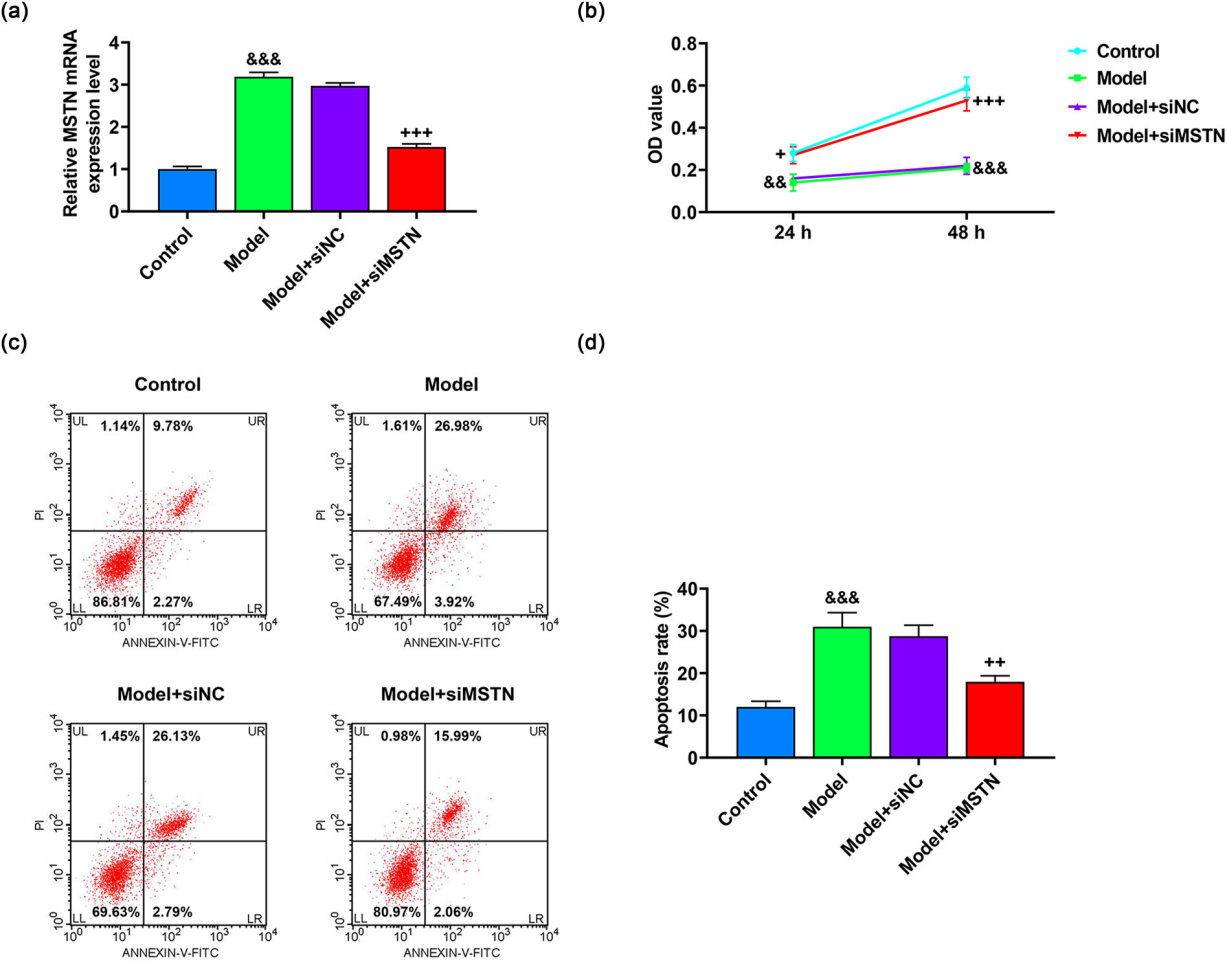
Effects of siMTSN on the viability and apoptosis of Ang II-induced AB8/13 cells. AB8/13 cells were treated with Ang II (100 nmol/L) to induce MN cell model, followed by the transfection of siNC or siMSTN. (a) Expression of MSTN in the control, model, model + siNC, and model + siMSTN groups was quantified by qRT-PCR, with GAPDH serving as the internal reference. (b) OD value of AB8/13 cells at 24 and 48 h was assessed by CCK-8 assay. (c and d) AB8/13 cell apoptosis was determined by flow cytometry. All experiments were repeated three times to obtain average values. The data are presented as the mean ± SD of three independent experiments; ^&&^ *p* < 0.01, ^&&&^ *p* < 0.001 vs Control; ^+^ *p* < 0.05; ^++^ *p* < 0.01; ^+++^ *p* < 0.001 vs Model + siNC. Abbreviation: MN, membranous nephropathy; MSTN, myostatin; Ang II, angiotensin II; qRT-PCR, quantitative real-time PCR; GAPDH, glyceraldehyde-3-phosphate dehydrogenase; OD, optical density; CCK-8, cell counting kit-8; siMSTN, small interfering RNA targeting MSTN; siNC, siRNA negative control.

**Figure A1:**
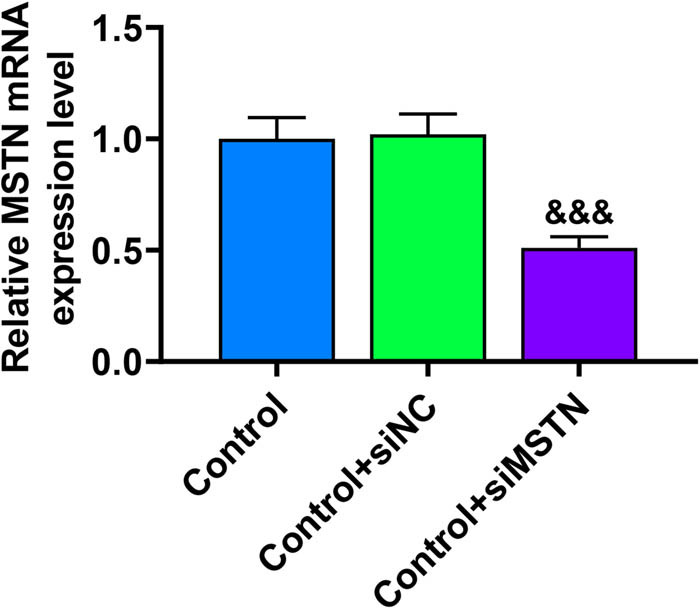
Effects of siMTSN on the expression of MTSN in AB8/13 cells. ^&&&^ *p* < 0.001 vs. control + siNC. Abbreviation: MSTN, myostatin; siMSTN, small interfering RNA targeting MSTN; siNC, siRNA negative control.

Next, siMSTN was transfected into AB8/13 cells and then resulted in the marked decrease in MSTN expression (*p* < 0.001, Figure A1), which evidenced the specificity of MSTN knockdown. To assess the function of MSTN in MN model cells, MSTN expression level was also knocked down in Ang II-induced AB8/13 cells. As depicted in Figure 3a, the expression of MSTN was largely increased in the model group, while being lessened in model + siMSTN group, indicating that the knockdown of MSTN suppressed the Ang II-induced MSTN expression (*p* < 0.001). After the modeling, Ang II stimulation prominently reduced the viability yet enhanced the apoptosis of AB8/13 cells (*p* < 0.01, Figure 3b–d). Based on the treatment of Ang II, silenced MSTN evidently increased the viability and suppressed the apoptosis of AB8/13 cells (*p* < 0.05, Figure 3b–d).

**Figure 4:**
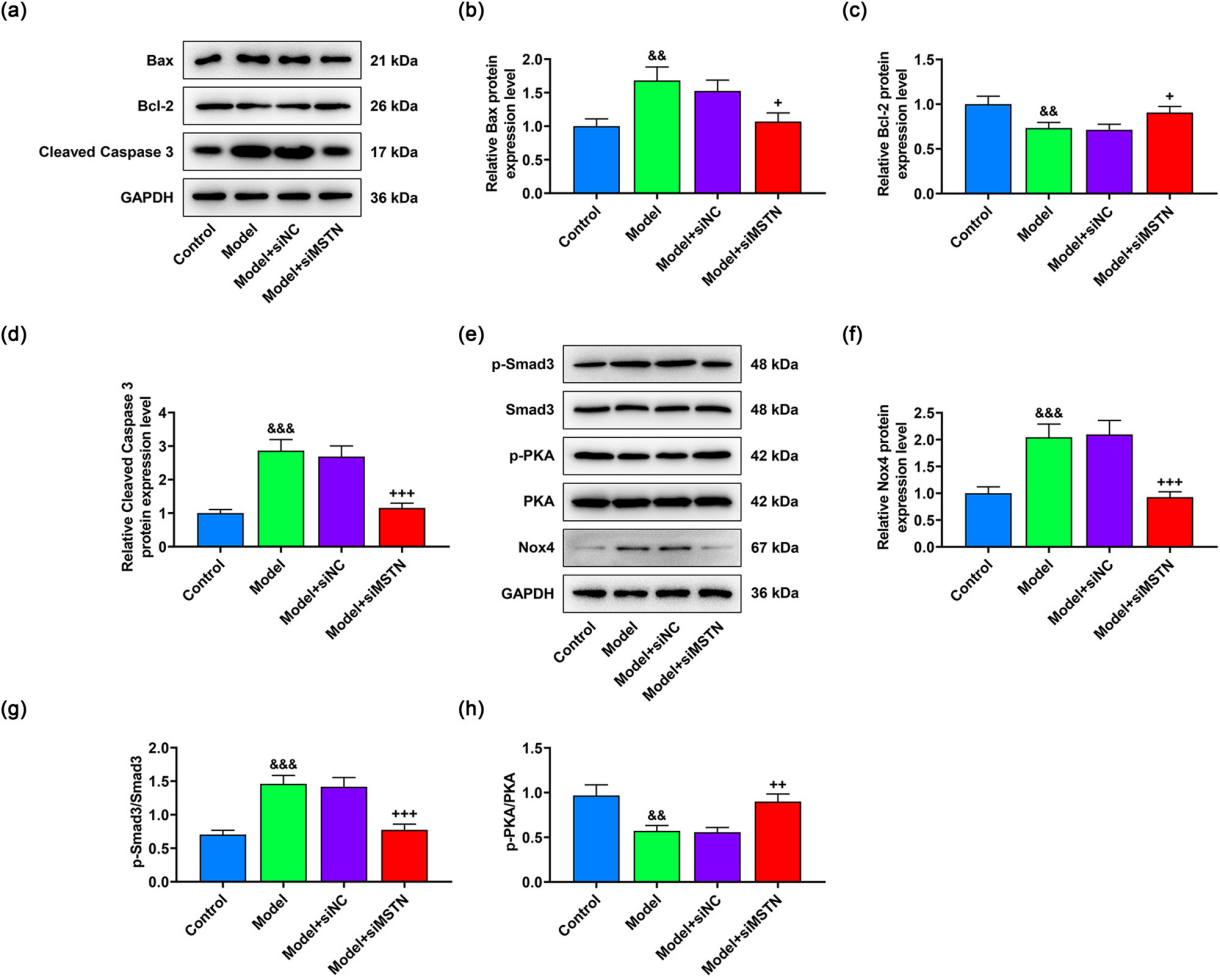
Effects of siMTSN on the protein expressions related to apoptosis and Smad3/PKA/Nox4 signaling pathway in Ang II-induced AB8/13 cells. AB8/13 cells were treated with Ang II (100 nmol/L) to induce MN cell model, followed by the transfection of siNC or siMSTN. (a–d) Expressions of apoptosis-related proteins (Bax, Bcl-2, and cleaved Caspase-3) in the control, model, model + siNC, and model + siMSTN groups were examined by western blot. GAPDH acted as the internal reference. (e–h) Expressions of Smad3/PKA/Nox4 signaling pathway-related proteins (p-Smad3, Smad3, p-PKA, PKA, and Nox4) were determined by western blot, with GAPDH serving as the internal reference. All experiments were repeated three times to obtain average values. The data are described as the mean ± SD of three independent experiments; ^&&^ *p* < 0.01, ^&&&^ *p* < 0.001 vs Control; ^+^ *p* < 0.05; ^++^ *p* < 0.01; ^+++^ *p* < 0.001 vs Model + siNC. Abbreviation: MN, membranous nephropathy; MSTN, myostatin; Ang II, angiotensin II; p-PKA, phosphorylated-protein kinase A; Nox4, NADPH oxidase 4; GAPDH, glyceraldehyde-3-phosphate dehydrogenase; siMSTN, small interfering RNA targeting MSTN; siNC, siRNA negative control.

Next, the protein expressions related to apoptosis and Smad3/PKA/Nox4 signaling pathway were detected to probe how MSTN silencing affected the viability and apoptosis of AB8/13 cells. Consequently, the increased expressions of Bax and cleaved Caspase-3 as well as the decreased expression of Bcl-2 that were induced by Ang II treatment were all reversely regulated by siMSTN (*p* < 0.05, Figure 4a–d). Similarly, lower levels of Nox4 expression and p-Smad3/Smad3 and higher level of p-PKA/PKA were observed in the model + siMSTN group than those in the model group (*p* < 0.01, Figure 4e–h). The above data illustrated that MSTN silencing may alleviate Ang II-induced podocyte injury through inhibiting the activation of Smad3/PKA/Nox4 signaling pathway.

### 3.3 Smad3 overexpression reversed the effects of siMSTN on the viability, apoptosis, and PKA/Nox4 signaling pathway in AB8/13 cells

**Figure 5:**
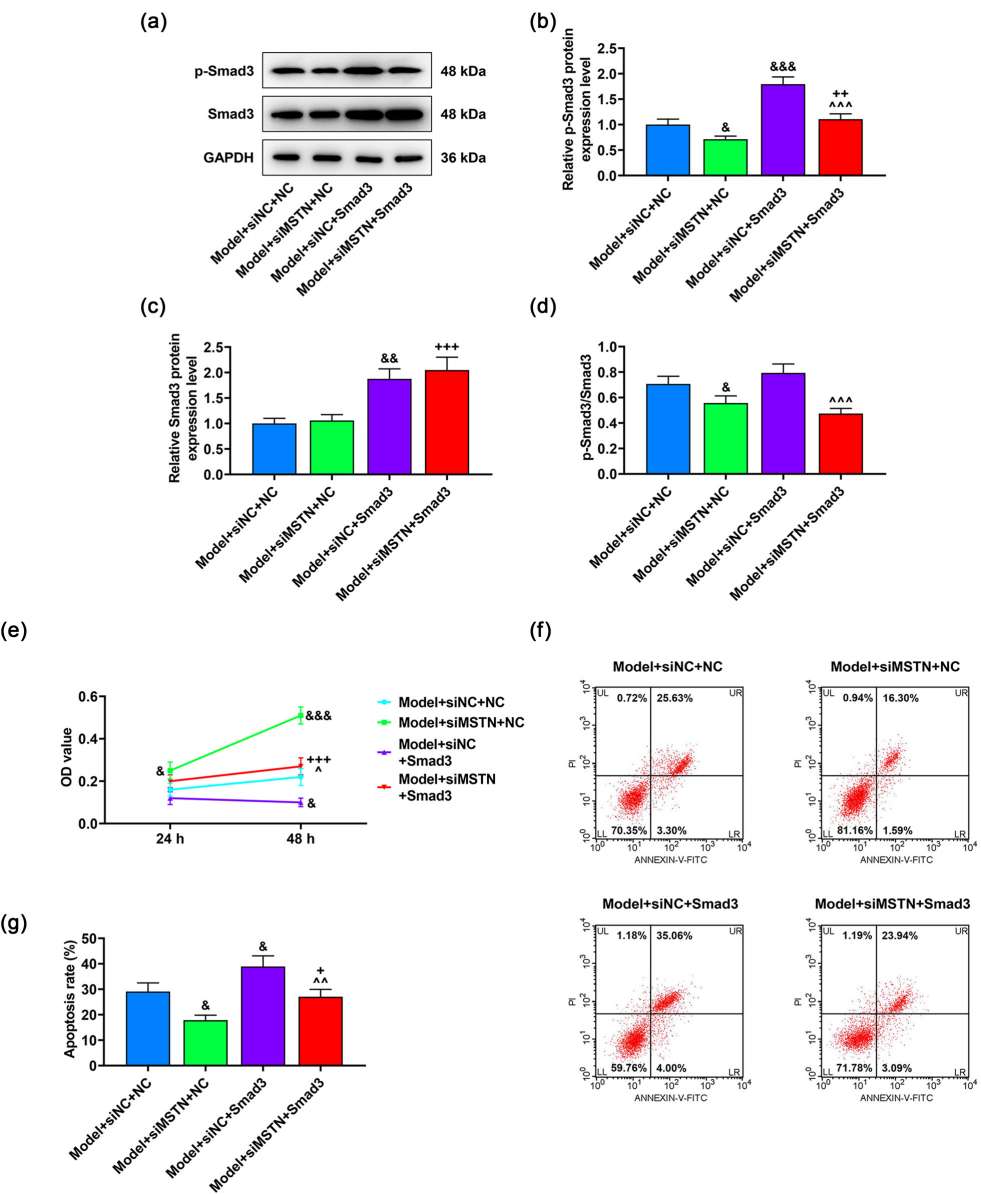
Effects of overexpressed Smad3 on the viability and apoptosis of MN model cells. AB8/13 cells were treated with Ang II (100 nmol/L) to induce MN cell model, followed by the transfection of siNC or siMSTN and NC or Smad3 overexpression plasmid. (a–d) Protein expressions of p-Smad3 and Smad3 as well as p-Smad3/Smad3 ratio were detected by western blot. GAPDH functioned as the internal reference. (e) CCK-8 assay was employed to measure the cell viability after the transfection and modeling. (f and g) Flow cytometry was applied to test the apoptosis of AB8/13 cells after the transfection and modeling. All experiments were repeated three times to obtain average values. The data are exhibited as the mean ± SD of three independent experiments; ^&^ *p* < 0.05, ^&&^ *p* < 0.01, ^&&&^ *p* < 0.001 vs Model + siNC + NC; ^+^ *p* < 0.05; ^++^ *p* < 0.01; ^+++^ *p* < 0.001 vs Model + siMSTN + NC; ^^^^ *p* < 0.01, ^^^^^ *p* < 0.001 vs Model + siNC + Smad3. Abbreviation: MN, membranous nephropathy; MSTN, myostatin; GAPDH, glyceraldehyde-3-phosphate dehydrogenase; CCK-8, cell counting kit-8; siMSTN, small interfering RNA targeting MSTN; siNC, siRNA negative control.

To further uncover the interaction between MSTN and Smad3 in MN model cells, we overexpressed Smad3 in AB8/13 cells and found that Smad3 overexpression promoted the expressions of p-Smad3 and Smad3 (*p* < 0.01, Figure 5a–d), which illustrated that the transfection was successful. Additionally, the cell viability was reduced but the apoptosis rate was induced in the model + siNC + Smad3 group in comparison with those in the model + siNC + NC group (*p* < 0.05, Figure 5e–g). Meanwhile, the impacts of MSTN silencing on increasing the cell viability and decreasing the cell apoptosis were negated by Smad3 overexpression (*p* < 0.05, Figure 5e–g).

**Figure 6:**
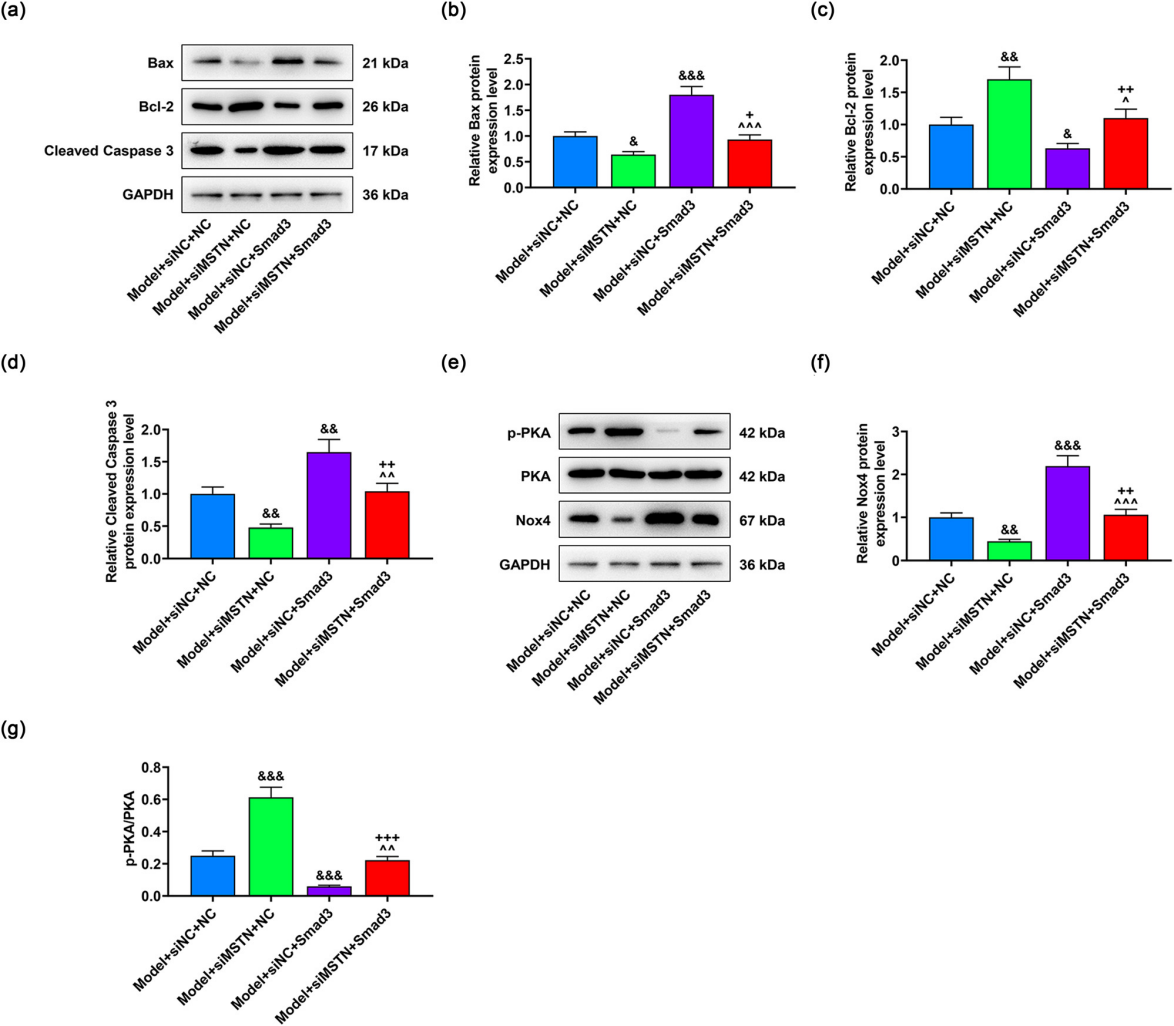
Effects of overexpressed Smad3 on the protein expressions related to apoptosis and PKA/Nox4 signaling pathway in MN model cells. AB8/13 cells were treated with Ang II (100 nmol/L) to induce MN cell model, followed by the transfection of siNC or siMSTN and NC or Smad3 overexpression plasmid. (a–d) Expressions of apoptosis-related proteins in the model + siNC + NC, model + siMSTN + NC, model + siNC + Smad3, and model + siMSTN + Smad3 groups were determined by western blot, with GAPDH serving as the internal reference. (e–g) After the transfection, the PKA/Nox4 signaling pathway-related protein expressions were detected by western blot, with GAPDH acting as the internal reference. All experiments were repeated three times to obtain average values. The data are displayed as the mean ± SD of three independent experiments; ^&^ *p* < 0.05, ^&&^ *p* < 0.01, ^&&&^ *p* < 0.001 vs Model + siNC + NC; ^+^ *p* < 0.05; ^++^ *p* < 0.01; ^+++^ *p* < 0.001 vs Model + siMSTN + NC; ^^^ *p* < 0.05, ^^^^ *p* < 0.01, ^^^^^ *p* < 0.001 vs Model + siNC + Smad3. Abbreviation: MN, membranous nephropathy; MSTN, myostatin; p-PKA, phosphorylated-protein kinase A; Nox4, NADPH oxidase 4; GAPDH, glyceraldehyde-3-phosphate dehydrogenase; siMSTN, small interfering RNA targeting MSTN; siNC, siRNA negative control.

Moreover, the downregulation of Bax and cleaved Caspase-3 and the upregulation of Bcl-2 induced by siMSTN was also counteracted by Smad3 overexpression (*p* < 0.05, Figure 6a–d). The similar effect was seen with overexpressed Smad3 on PKA/Nox4 signaling pathway. In detail, Smad3 overexpression neutralized the effect of siMSTN and promoted the expression of Nox4 but reduced p-PKA/PKA level (*p* < 0.01, Figure 6e–g). To conclude, overexpressed Smad3 could reverse the effects of siMSTN on regulating the viability, apoptosis, and PKA/Nox4 signaling pathway in AB8/13 cells.

### 3.4 MSTN silencing alleviated renal tissue injury and cell apoptosis in MN model rats via Smad3/PKA/Nox4 signaling pathway

**Figure 7:**
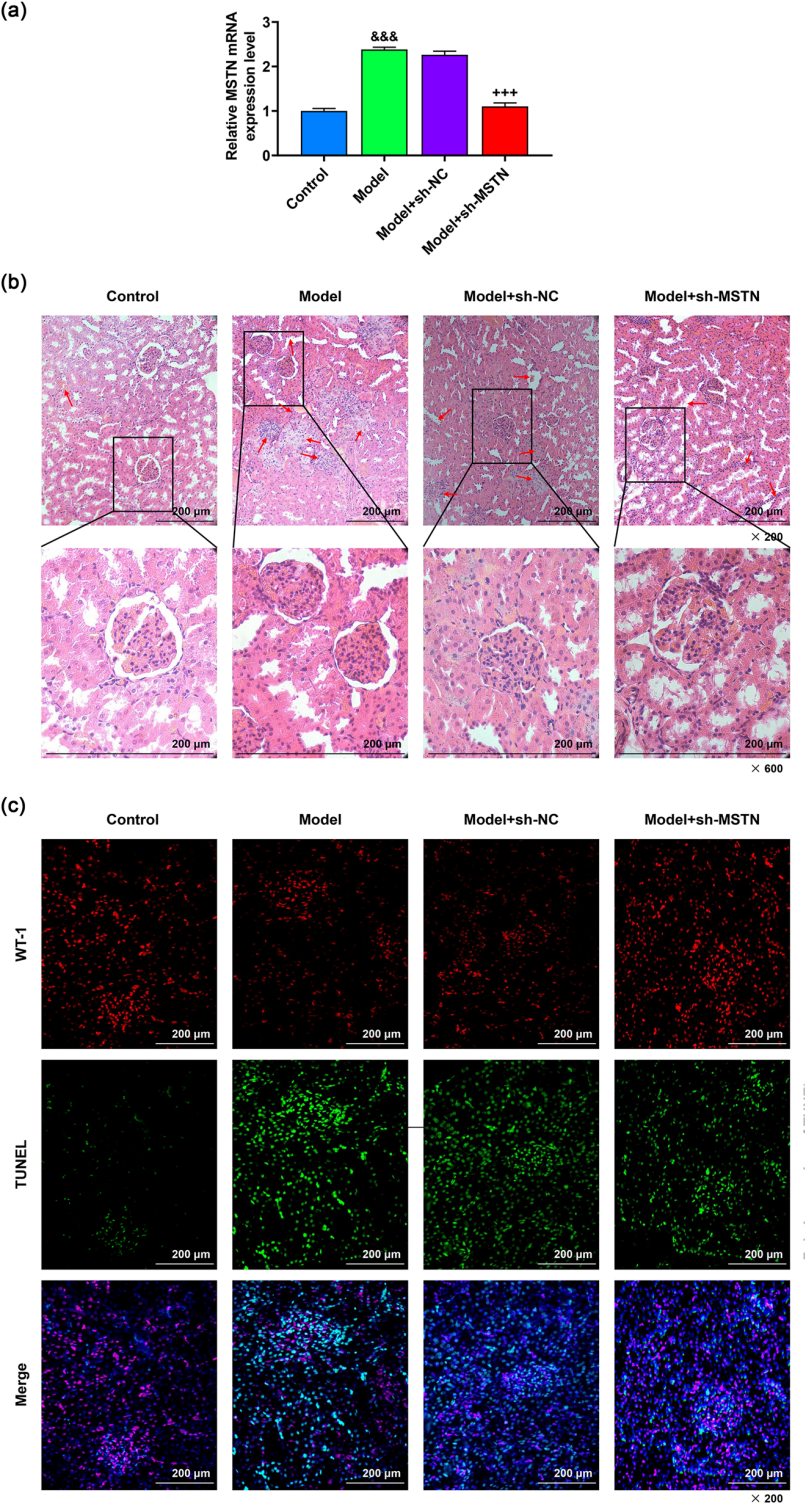
Effect of sh-MSTN on the kidney injury in MN model rats. MN model rats were induced by c-BSA and then injected with 30 pmol/g of sh-MSTN or sh-NC. (a) Expression of MSTN in kidney tissues of rats in the control, model, model+sh-NC and model+sh-MSTN groups was detected by qRT-PCR, with GAPDH serving as the internal reference. (b) H&E staining was adopted to evaluate the renal pathological status of rats in each group (magnification ×200, ×600, scale bar = 200 µm). (c) TUNEL assay was utilized to determine the apoptosis of rat renal cells in each group (magnification ×200, scale bar = 200 µm). All experiments were repeated three times to obtain average values. The data are expressed as the mean ± SD of three independent experiments; ^&&&^ *p* < 0.001 vs control; ^+++^ *p* < 0.001 vs Model+sh-NC. Abbreviation: MN, membranous nephropathy; MSTN, myostatin; GAPDH, glyceraldehyde-3-phosphate dehydrogenase; H&E, hematoxylin and eosin; TUNEL, terminal deoxynucleotidyl transferase-mediated dUTP nick end labeling; sh-MSTN, short hairpin RNA targeting MSTN; sh-NC, shRNA negative control.

As depicted in Figure 7a, there was a high expression of MSTN in the model group (*p* < 0.001, Figure 7a), while the treatment of sh-MSTN diminished the MSTN expression in model rats (*p* < 0.001, Figure 7a). The results of H&E staining assay mirrored that in the kidney tissues of model rats, the glomerulus was obviously swollen, the GBM was thickened, the capillary ring was compressed, the lumen was narrow or even closed, the balloon lumen became narrow or even adhesion occurred, mesangial cells and matrix proliferated, part of renal tubular epithelium was swollen, interstitial collagen fiber deposition was obvious, and the degree of fibrosis was high, while the renal tissue injury was dramatically mitigated in the rats of model + sh-MSTN group (Figure 7b). Concurrently, the number of TUNEL-positive rat renal cells was increased by a large margin after modeling (Figure 7c). Moreover, the podocytes stained with WT-1 were decreased in the model group as compared with those in the control group (Figure 7c). After the modeling, these changes in TUNEL-positive cells and WT-1-positive cells were remarkably reversed by sh-MSTN (Figure 7c).

**Figure 8:**
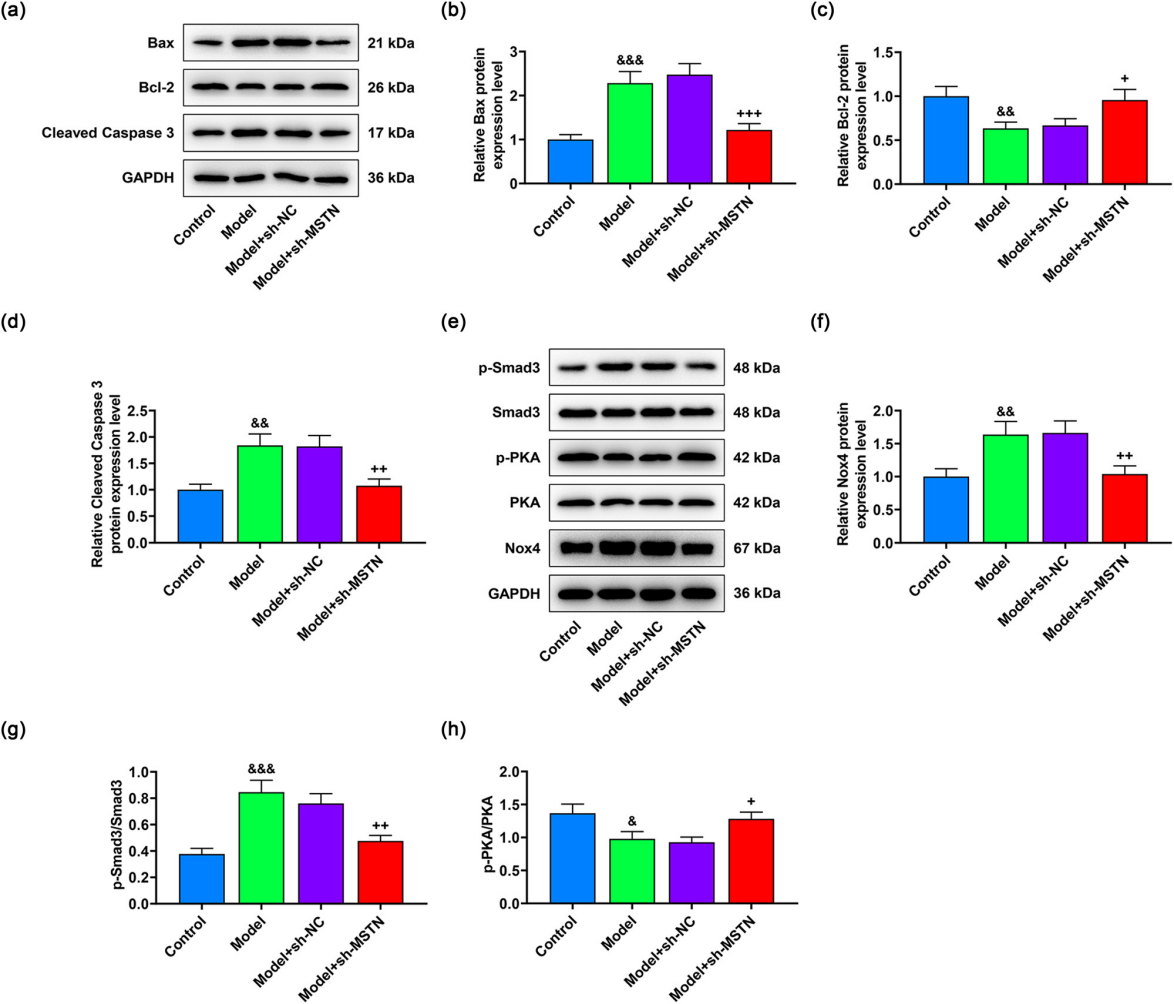
Effects of sh-MSTN on the apoptosis and expressions of Smad3/PKA/Nox4 signaling pathway-related proteins in MN model rats. MN model rats were induced by c-BSA and then subjected to injection with 30 pmol/g of sh-MSTN or sh-NC. (a–d) Expression levels of the apoptosis-related proteins in rat renal tissues were examined by western blot, with GAPDH acting as the internal reference. (e–h) Western blot was also performed to measure the expressions of Smad3/PKA/Nox4 signaling pathway-related proteins in rat renal tissues, with GAPDH functioning as the internal reference. All experiments were repeated three times to obtain average values. The data are presented as the mean ± SD of three independent experiments; ^&^ *p* < 0.05, ^&&^ *p* < 0.01, ^&&&^ *p* < 0.001 vs Control; ^+^ *p* < 0.05, ^++^ *p* < 0.01, ^+++^ *p* < 0.001 vs Model + sh-NC. Abbreviation: MN, membranous nephropathy; MSTN, myostatin; p-PKA, phosphorylated-protein kinase A; Nox4, NADPH oxidase 4; GAPDH, glyceraldehyde-3-phosphate dehydrogenase; sh-MSTN, short hairpin RNA targeting MSTN; sh-NC, shRNA negative control.

In addition, sh-MSTN had the reversal effect on the high expressions of Bax and cleaved Caspase-3 and low expression of Bcl-2 in the kidney tissues of model rats (*p* < 0.05, Figure 8a–d). The enhancement of Nox4 and p-Smad3/Smad3 levels, together with the inhibition of p-PKA/PKA level, was observable in model rat kidney tissues (*p* < 0.05, Figure 8e–h), whereas sh-MSTN offset these alterations of protein expression levels (*p* < 0.05, Figure 8e–h). All in all, MSTN silencing alleviated renal tissue injury and cell apoptosis in MN model rats through blocking Smad3/PKA/Nox4 signaling pathway.

## 4 Discussion

MN is a glomerular disease caused by multiple etiologies, and a decrease in podocyte number is a contributing factor to the pathogenesis of MN, while podocyte apoptosis can lead to podocyte decrease, signifying that podocyte apoptosis is related to the occurrence of MN [21]. Through the bioinformatics analyses, we found the strong correlation between the upregulated genes in GSE73953 dataset and apoptosis, and MSTN was the most apparently upregulated gene. Accordingly, we speculated that MSTN may participate in the pathological process of MN by regulating podocyte apoptosis. Our experimental results proved that MSTN silencing hindered the apoptosis in MN model cells and rats, and alleviated the rat kidney injury, confirming its potential as a target for the treatment of MN.

MSTN belongs to the transforming growth factor-β (TGF-β) superfamily, TGF-β is currently known as the most predominant factor leading to fibrosis, which is involved in the regulation of cell growth, glomerular mesangial cell proliferation, and extracellular matrix formation [22]. Smad protein family is directly involved in the signal transduction of TGF-β superfamily members and is the initiating factor in the intracellular signaling of TGF-β, while activated TGF-β receptors can regulate the transcription of substances in the nucleus through Smad 2, 3, and 4 [23]. It has been proved that negative auto-regulation of MSTN expression is mediated by Smad3 [24], and long noncoding RNA TSI could inhibit renal fibrogenesis through negatively regulating the TGF-β/Smad3 pathway [25]. In this study, we found that the protein expression of p-Smad3 was activated in model cells, while MSTN silencing inhibited the activation of p-Smad3, which was consistent to the results in the study by Retamales et al. [14]. Therefore, MSTN silencing may repress the podocyte apoptosis in MN model via regulating Smad3 and its downstream pathways, while this mechanism has never been revealed before.

It has been elucidated that TGF-β is one of the targets of cyclic adenosine monophosphate (cAMP)/PKA/cAMP-responsive element binding protein (CREB) signaling pathway [26]. As previously documented, upregulation of cAMP inhibits fibroblast proliferation, blocks the transformation of AngII/TGF-β1-induced fibroblast to myofibroblasts, and reduces extracellular matrix deposition [27]. PKA is the key downstream target of cAMP, and activated PKA can phosphorylate serine 133 (Ser133) of CREB [28]. Besides, p-CREB can competitively bind to CREB binding protein with the Smad complex, leading to the decrease in the level of p-Smad3 [29,30]. In addition, NOX is a kind of NADPH oxidase homolog that consists of seven different subunits (Nox1, NOX2, NOX3, NOX4, NOX5, Duox1, and Duox 2), which is an important inducer of oxidative stress [31]. In rat podocytes, NOX4 is mainly located in the mitochondria, and the mitochondrial membrane potential is signally depolarized by the TGF-β1-mediated upregulation of NOX4. TGF-β1 can cause an increase in NOX4 expression, ROS generation, loss of mitochondrial membrane potential, and caspase-3 activation, while these effects of TGF-β1 can be offset by knockdown of either Smad2 or Smad3 [32]. Also, the study of Guo et al. mentioned that Smad3 could upregulate the expression of Nox4 and suppress PKA activity to regulate the podocyte apoptosis triggered by HG [15]. Similar to the previous literature, our research revealed that knockdown of MSTN reduced the expression of Nox4 but augmented p-PKA/PKA level in both MN cells and rat models, whereas Smad3 overexpression reversed its function and contributed to the upregulation of Nox4 and the decline of p-PKA/PKA. These results implied that the MSTN knockdown exerted its anti-apoptotic effect through the Smad3/PKA/Nox4 pathway. Notably, our study was the first to elucidate the association between MSTN and Smad3/PKA/Nox4 signaling pathway in the progression of MN.

Besides, the occurrence of apoptosis is usually regulated by Bcl-2 and Bax inside the mitochondria, in which Bcl-2 can prevent the release of cytochrome c into the cytosol to inhibit apoptosis, whereas the effect of Bax is exactly the opposite [33]. Caspase-3 is the final executor in the process of apoptosis, and its activation can directly lead to cell apoptosis [34]. The results of western blot in the present study demonstrated that Bax and cleaved Caspase-3 expressions were upregulated, while the expression of Bcl-2 was downregulated in Ang-II-stimulated podocytes, suggesting the promotion of apoptosis in model cells. Moreover, MSTN knockdown hindered the apoptosis by reversely regulating these protein expressions, and Smad3 upregulation was able to countervail its effect.

Collectively, MSTN silencing mitigates the podocyte apoptosis in MN models by modulating Smad3/PKA/Nox4 pathway, hinting its potential as an indicator in molecular targeted therapy of MN. Nevertheless, only one podocyte cell line was selected in this article, and more podocyte cell lines will be chosen in our future study.
